# Competing Biases in Real-Time Sentence Processing

**DOI:** 10.5334/joc.507

**Published:** 2026-07-01

**Authors:** Hiroki Fujita

**Affiliations:** 1 Department of Linguistics, University of Potsdam, Potsdam, Germany

**Keywords:** Ambiguity resolution, constraint-based approach, sentence processing, parsing, computational modelling

## Abstract

In sentences such as “*John remembered the boy took some time to rest*”, the locally ambiguous noun phrase (NP) “*the boy*” is initially parsed as the direct object of the matrix verb “*remembered*” (the object analysis). When the embedded verb “*took*” is encountered, the NP is revised as the subject of the embedded clause (the subject analysis). Open questions are how the real-time resolution of this complement ambiguity is influenced by semantic/categorial constraints (i.e., whether the locally ambiguous NP is a semantically/categorially appropriate object of the matrix verb) and selectional frequency (i.e., the frequency with which the matrix verb takes a direct object NP). The present study addressed these questions and also examined whether temporal adjuncts, which can bias parsing towards the object analysis (e.g., “*John remembered the boy after…*”), influence real-time ambiguity resolution. The results showed that semantic/categorial constraints and selectional frequency drive the processor towards the subject analysis before embedded-verb disambiguation. However, temporal adjuncts gradually increased in influence and ultimately overrode these biases during processing. The observed parsing process was also simulated within an interactive constraint-based framework. Together, the experimental and simulation results suggest that real-time sentence processing is dynamically shaped by multiple competing biases.

## Introduction

When reading a sentence, readers can often arrive at more than one interpretation until disambiguating information becomes available. A central question in sentence-processing research concerns how such local ambiguities are resolved during real-time processing (e.g., Adams et al., 1998; Clifton et al., 2003; Clifton, 1993; Ferreira & Clifton, 1986; Ferreira & Henderson, 1990; Fodor & Ferreira, 1998; L. Frazier, 1979; Fujita, 2026; Fujita & Yoshida, 2026b; Garnsey et al., 1997; Kennison, 2001; McRae et al., 1998; Mitchell, 1987; Omaki et al., 2015; Pickering et al., 2000; Pickering & Traxler, 2003; Qian et al., 2019; Sturt, 1997; Trueswell et al., 1993; van Gompel & Pickering, 2001). The present study investigates the real-time resolution of one type of local ambiguity—complement ambiguity—which arises in sentences like (1).

(1)    John remembered the boy took some time to rest.

**Figure 1:**
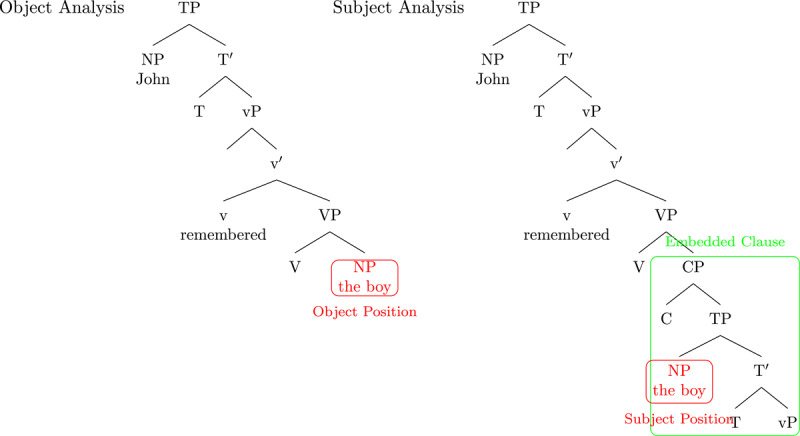
Object and subject analyses in (1) “John remembered the boy…”.

In (1), the noun phrase (NP) “*the boy*” is locally ambiguous between two analyses: it can be parsed either as the direct object of the matrix verb “*remembered*” (“*John remembered the boy*”; the object analysis) or as the subject of the embedded clause (“*John remembered* that *the boy…*”; the subject analysis). These two analyses are illustrated in Figure 1.

In (1), the object analysis becomes unavailable at the embedded verb “*took*”, because this verb requires “*the boy*” to serve as the subject of the embedded clause. Substantial evidence suggests that the object analysis is initially preferred when the locally ambiguous NP is a semantically and categorially appropriate object of the matrix verb and when the matrix verb frequently occurs with this analysis (e.g., L. Frazier, 1979; Fujita, 2021; Gibson, 1991; Gorrell, 1995; Pritchett, 1992; Sturt et al., 1999; Traxler & Pickering, 1996; Trueswell et al., 1993). For example, in (1), “*the boy*” is a semantically plausible object of “*remembered*” and belongs to an NP category that the verb can take as its object, and “*remembered*” frequently takes a direct object NP (e.g., Ferreira & Henderson, 1990; Garnsey et al., 1997; Holmes et al., 1989; Pickering & Traxler, 1998, 2003; Schmauder & Egan, 1998; Sturt et al., 1999; Trueswell et al., 1993).

Evidence for an initial preference for the object analysis comes from studies reporting processing difficulty at the disambiguating region in sentences like (1) (e.g., L. Frazier & Rayner, 1982; Fujita, 2021; Rayner & Frazier, 1987; Sturt et al., 1999). This difficulty is often referred to as a garden-path effect (e.g., L. Frazier & Rayner, 1982; Fujita, 2024b; Pritchett, 1988, 1992). Previous research shows that reading times increase at the disambiguating verb “*took*” in (1), relative to sentences without complement ambiguity, as in (2).

(2)    John remembered that the boy took some time to rest.

Sentence (2) is identical to (1) except for the presence of the overt complementiser “*that*”, which prevents the locally ambiguous NP “*the boy*” from being parsed as the object of “*remembered*”. Increased reading time at “*took*” in (1)—a garden-path effect—indicates that when “*the boy*” is encountered during real-time processing, it is parsed as the object of “*remembered*”, and this object analysis is maintained until disambiguation at “*took*” (Ferreira & Henderson, 1990; Rayner & Frazier, 1987; Sturt et al., 1999; Trueswell et al., 1993).

The object analysis is initially preferred in (1). However, it remains debated whether this preference persists when factors such as semantic/categorial constraints and selectional frequency bias parsing towards the subject analysis. Pickering et al. (2000), for example, examined sentences like (3a/b) to test whether some of these factors override the processor’s preference for the object analysis.

(3a)    The athlete realised her potential might make her famous.
(3b)    The athlete realised her exercises might make her famous.

In (3a/b), the matrix verb is “*realised*”, which often occurs with the subject analysis (e.g., Trueswell et al., 1993). In (3a), the locally ambiguous NP is a semantically plausible object of “*realised*” (“*The athlete realised her potential*”), whereas in (3b), it is a semantically implausible object (“*The athlete realised her exercises”). If the processor ignores these biases and analyses the locally ambiguous NP as the direct object of the matrix verb, reading times should be longer at “*her exercises*” in (3b) than at “*her potential*” in (3a) because the semantically implausible object “*her exercises*” should cause interpretive difficulty (e.g., Fujita & Cunnings, 2022; Phillips, 2006; Pickering & Traxler, 1998). In a reading experiment, Pickering et al. observed this pattern, suggesting that the processor adopts the object analysis at the locally ambiguous NP even when semantic and frequency-based selectional biases favour the subject analysis.

The present study examines whether the object analysis is maintained until the embedded verb appears even when semantic/categorial constraints and selectional frequency favour the subject analysis, or whether these biases lead to adoption of the subject analysis before disambiguation (Research Question 1). Previous work has yielded mixed findings on this issue (e.g., Ferreira & Henderson, 1990; Garnsey et al., 1997; Holmes et al., 1989; Trueswell et al., 1993). The study also investigates whether temporal adjuncts affect real-time ambiguity resolution and how they interact with semantic/categorial constraints and selectional frequency (Research Question 2). Each question is addressed in turn below.

As mentioned above, findings for the first research question are mixed. Ferreira and Henderson (1990), for instance, investigated how complementiser-absent sentences like (3b) are processed relative to complementiser-present sentences such as “*The athlete realised that her exercises might…*”. In a reading experiment, Ferreira and Henderson reported a garden-path effect at the embedded verb in complementiser-absent sentences. This finding suggests that the object analysis is computed at the locally ambiguous NP and maintained until the embedded verb is encountered, even when semantic/categorial constraints and selectional frequency favour the subject analysis (see also Holmes et al., 1989).

By contrast, Garnsey et al. (1997) reported no garden-path effect when semantic constraints and selectional frequency favoured the subject analysis. Garnsey et al. argue that this finding supports the interactive constraint-based model, which posits that multiple biases begin influencing real-time processing immediately after they become available (e.g., McRae et al., 1998; McRae & Matsuki, 2013; Spivey-Knowlton & Sedivy, 1995; Spivey-Knowlton & Tanenhaus, 1998; Tanenhaus et al., 2000).

In the constraint-based model, each bias contributes to processing via activation levels, and a preferred analysis emerges through a three-stage normalised recurrence process (Spivey-Knowlton, 1996). Specifically, for each bias *b* and analysis *a*, the activation *S* is normalised as follows: $S_{\text{b,a}}^{\prime }=\frac{S_{\text{b,a}}}{\Sigma_{a}S_{\text{b,a}}}$ , where the activation for an analysis is divided by the sum of activations for all competing analyses within a given bias. The normalised activations are then combined for each analysis via a weighted sum: $i_{a}=\Sigma_{b}w_{b}S_{\text{b,a}}^{\prime }$ . By default, the weights (*w_b_*) are equal; for example, with three biases, each contributes: $\frac{S_{\text{b,a}}^{\prime }}{3}$ . Activations are then updated based on the normalised activation, combined activation, and weight: $S_{\text{b,a}}=S_{\text{b,a}}^{\prime }+i_{a}w_{b}S_{\text{b,a}}^{\prime }$ . This cycle repeats until one analysis reaches a threshold T=1−xy , where *x* is a constant, and *y* is the number of cycles. The analysis that reaches *T* is selected by the processor.

**Figure 2:**
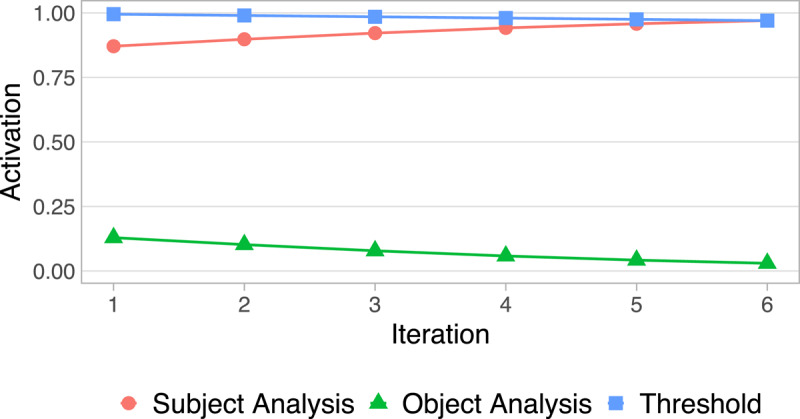
Constraint-based model simulation for sentence (3b).

To illustrate, Trueswell et al. (1993) reported that the verb “*realised*” co-occurs with the subject analysis (SA) with a probability *p* = .93 relative to the object analysis (OA). Pickering et al. (2000) reported that, on a plausibility scale from 1 (*implausible*) to 7 (*plausible*), “*her exercises*” as the direct object of “*realised*” received a rating *r* ≈ 2, whereas the subject analysis with “*her exercises*” as the embedded subject received a rating of about 6. First, these input values are normalised: $S_{p,Sa}^{\prime }=.93,\;S_{p,Oa}^{\prime }=.07$ (at the matrix verb: “*The athlete **realised**…*”) and $S_{r,Sa}^{\prime }=.75,\;S_{r,Oa}^{\prime }=.25$ (at the locally ambiguous NP: “*The athlete realised **her exercises**…*”). Assuming equal weights, the combined activation values are: *i_SA_* ≈ .87 and *i_OA_* ≈ .13. Subsequently, the strengths of the biases are updated and normalised, giving: $S_{p,Sa}^{\prime }\approx .98,\;S_{p,Oa}^{\prime }\approx .02$ and $\;S_{r,Sa}^{\prime }\approx .80,\;S_{r,Oa}^{\prime }\approx .20$ . With the activation threshold constant set to 005, *i_SA_* reaches the threshold quickly, as illustrated in Figure 2. Because the constraint-based model predicts an immediate influence of available biases on real-time ambiguity resolution, the results of Garnsey et al. (1997) are consistent with this model.

Now, I turn to the second research question: whether temporal adjuncts affect the real-time resolution of complement ambiguity and how they interact with semantic/categorial constraints and selectional frequency. Temporal adjuncts are adjuncts headed by prepositions such as “*after*” and “*before*”. Like adverbs, they can occupy multiple positions within a sentence (e.g., Green, 2018; Landau, 2021) and, when placed immediately after a locally ambiguous NP, may introduce a strong bias towards the object analysis. Consider (4):

(4)    The union leader implied the raise after…

In (4), “*the raise*” is locally ambiguous and is a semantically/categorially appropriate object of the matrix verb “*implied*”. Crucially, the temporal adjunct “*after…*” immediately follows “*the raise*”. As noted, temporal adjuncts are distributionally flexible, and the position of the temporal adjunct in (4) depends on how “*the raise*” is interpreted. Specifically, if “*the raise*” is parsed as the object of “*implied*”, “*after…*” is placed post-verbally and modifies the matrix verb “*implied*”:

(5)    The union leader implied the raise after negotiations concluded.

However, if “*the raise*” is parsed as the subject of the embedded clause, “*after…*” is integrated within that embedded clause:

(6)    The union leader implied that the raise, after negotiations concluded, would happen.

The temporal adjunct is expected to strongly bias parsing towards the object analysis, given that the postverbal position in (5) is far more frequent than the medially adjoined position in (6). To test this hypothesis, I used GPT-2 (Radford et al., 2019) to estimate the relative likelihood of continuations consistent with the subject or object analysis for (4) (adjunct-present), and for a version of (4) with “*after*” removed (adjunct-absent). GPT-2 estimates the predictability of text by assigning a probability to each word given its preceding context: $P(w_{j}|w_{<j})$ . Therefore, GPT-2 can be used to sample likely continuations based on the preceding words.

Using GPT-2, I generated 100 continuations for each of the adjunct-present and adjunct-absent conditions and classified the resulting sentences as reflecting either the subject or the object analysis. In these materials, “*implied*” serves as the matrix verb; previous research suggests that this verb frequently occurs with the subject analysis (Garnsey et al., 1997; Trueswell et al., 1993). Consistent with this research, in the adjunct-absent condition (“*The union leader implied the raise…*”), 99 out of 100 continuations reflected the subject analysis. By contrast, in the adjunct-present condition (“*The union leader implied the raise after…*”), 98 continuations reflected the object analysis. This pattern indicates that adjunct-present sentences like (4) strongly bias parsing towards the object analysis.^1^ The present study investigates whether, and how, this adjunct-induced bias influences real-time ambiguity resolution when semantic/categorial constraints and selectional frequency favour the subject analysis.

In summary, the present study addresses the following research questions:

Question 1: Is the object analysis maintained until the embedded verb appears when semantic/categorial constraints and selectional frequency favour the subject analysis?

Question 2: How does the adjunct bias interact with semantic/categorial constraints and selectional frequency during real-time ambiguity resolution?

In the following section, I describe the main research design used to address these questions.

## The present study

I conducted three reading experiments (Experiments 1–3) to investigate how semantic/categorial constraints, selectional frequency, and temporal adjuncts influence real-time ambiguity resolution. To address the research questions described in the previous section, I used a design that tests how the critical NP (“*the boy/girl*”) is processed, employing two diagnostic measures: (i) a garden-path effect at the embedded region (“*took*”) and (ii) a gender-mismatch effect—processing difficulty caused by mismatched gender—at a reflexive pronoun:

(7)    John hoped the boy/girl after washing himself in the bathroom took some time to rest from studying.

In (7), the matrix verb is “*hoped*”, which often occurs with the subject analysis (Trueswell et al., 1993), and the critical NP “*the boy/girl*” is not a semantically/categorially appropriate object of this verb. Thus, semantic/categorial constraints and selectional frequency favour the subject analysis. Crucially, “*the boy/girl*” is followed by a temporal adjunct containing a reflexive.

A reflexive is in general referentially dependent on another NP that locally c-commands it (e.g., Büring, 2005; Chomsky, 1981, 1982; Truswell, 2014). C-command is a structural relation between nodes: node x c-commands node y if x is a sister of y or x is a sister of node z that dominates y (Reinhart, 1976).

In (7), the NP that locally c-commands the reflexive is PRO—an unpronounced pronoun that serves as the subject of the temporal adjunct (“after **PRO** washing himself”) (e.g., Baltin, 1995; Boeckx et al., 2010; Chomsky, 1981; Green, 2018; Kawasaki, 1993; Landau, 2013, 2025; Martin, 2001; Reed, 2014; Williams, 1992). Thus, the reflexive and PRO are referentially linked: “*after [_NPk_ PRO] washing [_NPk_ himself]*”, where the co-indices *k* indicate coreference. Like reflexives, PRO is referentially dependent on another NP. Crucially, PRO’s preferred antecedent in (7) depends on whether the object or subject analysis is adopted, because the interpretation determines the position of the temporal adjunct (as illustrated in the previous section).

If the object analysis is adopted, the temporal adjunct is integrated into the matrix clause, and PRO (and hence the reflexive) preferentially corefers with the matrix subject NP “*John*”: “*[_NPk_ John] hoped the boy/girl after [_NPk_ PRO] washing [_NPk_ himself]…*” (compare “*John found the boy/girl after washing himself…*”). By contrast, if the subject analysis is adopted, the temporal adjunct is integrated into the embedded clause, making the NP “*the boy/girl*” a preferred antecedent for PRO: “*John hoped that [_NPk_ the boy/girl] after [_NPk_ PRO] washing [_NPk_ himself]…*”.

As mentioned above, gender-mismatch effects and garden-path effects were used as diagnostics to determine which analysis is adopted. In (7), the matrix subject NP (“*John*”) matches the reflexive (“*himself*”) in gender, whereas the critical NP either matches (“*the boy*”) or mismatches (“*the girl*”) the reflexive’s gender. Substantial evidence suggests that gender-mismatch effects arise during real-time sentence processing (e.g., M. Frazier et al., 2015; Fujita, 2023; Fujita & Yoshida, 2024; Giskes & Kush, 2021; González Alonso et al., 2021; Hall & Yoshida, 2021; Kazanina et al., 2007; Kush et al., 2017; Sturt, 2003; Yoshida et al., 2013).

On this basis, if the subject analysis is adopted due to semantic/categorial constraints and selectional frequency, a gender-mismatch effect should emerge at the reflexive when the critical NP mismatches its gender (i.e., increased processing difficulty should occur at “*himself*” in “*the girl…himself*”), and no garden-path effect is expected at the embedded verb “*took*”. By contrast, if the object analysis is adopted (due to the temporal adjunct), no gender-mismatch effect is expected at “*himself*” (because the reflexive corefers with the gender-matching matrix subject NP; “*John…himself*”), but a garden-path effect should occur at “*took*”.

Experiment 1 used the design illustrated in (7). Experiment 2 tested a similar design, but without the temporal adjunct, to examine whether semantic/categorial constraints and selectional frequency lead the processor to adopt the subject analysis before disambiguation at the embedded verb in the absence of adjunct-induced bias. Experiment 3 compared sentences with and without temporal adjuncts to directly assess their influence on real-time ambiguity resolution.

## Experiment 1

Experiment 1 employed the design illustrated in (8a–d) to examine how semantic/categorial constraints, selectional frequency, and temporal adjuncts influence real-time ambiguity resolution.

(8a)    *Complementiser absent, Gender match*

John hoped the boy after washing himself in the bathroom took some time to rest from studying.

(8b)    *Complementiser absent, Gender mismatch*

John hoped the girl after washing himself in the bathroom took some time to rest from studying.

(8c)    *Complementiser present, Gender match*

John hoped that the boy after washing himself in the bathroom took some time to rest from studying.

(8d)    *Complementiser present, Gender mismatch*

John hoped that the girl after washing himself in the bathroom took some time to rest from studying.

In (8a–d), the matrix verb is “*hoped*”, which often occurs with the subject analysis (Trueswell et al., 1993), and the critical NP “*the boy/girl*” is not a semantically/categorially appropriate object of this verb. In (8c/d), the overt complementiser “*that*” follows the matrix verb (“*John hoped that the…*”), whereas in (8a/b), it does not (“*John hoped the…*”). All conditions have a temporal adjunct containing a reflexive (“*after washing himself in the bathroom*”). The reflexive matches the critical NP’s gender (“*the boy/girl*”) in (8a/c) but mismatches it in (8b/d). In all conditions, the matrix subject NP (“*John*”) matches the reflexive’s gender.

If the object analysis is adopted at “*the boy/girl*” and maintained (due to the temporal adjunct), no gender-mismatch effect is expected at the reflexive in the complementiser-absent conditions (8a/b): under the object analysis, PRO (and hence the reflexive) is preferentially interpreted as coreferential with the matrix subject NP “*John*”, which matches it in gender: “*[_NPk_ John] hoped the boy/girl after [_NPk_ PRO]] washing [_NPk_ himself]…*”. In addition, a garden-path effect is expected at the embedded verb “*took*” in the complementiser-absent conditions.

By contrast, if the subject analysis is adopted and maintained due to semantic/categorial constraints and selectional frequency, a gender-mismatch effect should appear at the reflexive in both the complementiser-absent and complementiser-present conditions: under the subject analysis, “*the boy/girl*” is preferentially interpreted as the antecedent for PRO, which corefers with the reflexive: “*John hoped* that *[_NPk_ the boy/girl] after [_NPk_ PRO]] washing [_NPk_ himself]…*”. Furthermore, no garden-path effect is expected at “*took*” in the complementiser-absent conditions.

### Participants

In Experiment 1, 216 participants were recruited via Prolific (https://www.prolific.com/). All participants were native speakers of English, British citizens, university graduates, and reported having lived primarily in the UK before the age of 18. The experiments reported here were conducted with large samples, following recent work in sentence processing (e.g., Fujita, 2024a, 2025a; Huang et al., 2024; Jäger et al., 2020; Parker, 2019; Vasishth et al., 2018). The study was approved by the University of Reading Research Ethics Committee.

### Materials

The experiment included 24 sets of experimental sentences exemplified in (8a–d), 72 filler sentences, and practice items.

To ensure that the matrix verbs were biased towards the subject analysis, I selected subject-analysis-biased verbs from prior work (e.g., Trueswell et al., 1993). Semantic/categorial constraints were confirmed in a plausibility norming study. In this study, 40 native speakers of English (drawn from the same participant pool as Experiment 1, but not taking part in the main reading task) rated plausibility for truncated experimental sentences such as “*John hoped the boy*” and “*John hoped the girl*” on a 7-point scale (1 = *highly implausible*, 7 = *highly plausible*). The norming study also included 32 filler items (28 plausible, 4 implausible). Given that the experimental items were intended to be implausible, the norming materials as a whole were balanced such that half of the items were plausible and half were implausible. The experimental items received a mean plausibility rating of 1.9 (SD = 1.5), confirming that the critical NP was implausible as the direct object of the matrix verb.

### Procedure

Reading times were measured using an L-maze task (Boyce et al., 2020; Forster et al., 2009; Fujita, 2025b; Witzel et al., 2012) implemented in PCIbex Farm (Zehr & Schwarz, 2018). In this task, each sentence was presented one word at a time together with a pseudoword. Participants were instructed to select the word that correctly continued the sentence by pressing the “E” key for the left option or the “I” key for the right option. If a pseudoword was selected, the trial terminated immediately. The experiment began with practice trials, followed by 24 experimental sentences and 72 filler sentences presented in a pseudorandom order.

### Data analysis

Log-transformed reading times were analysed at four regions: the reflexive region (“*himself*”), the post-reflexive region (“*in*”), the verb region (“*took*”), and the post-verb region (“*some*”) regions. Before analysing the data, raw reading times below 200 milliseconds or above 6,000 milliseconds were excluded as likely reflecting attentional lapses. Linear mixed-effects models were fitted using lme4 (Bates et al., 2015) in R (R Core Team, 2025). The predictor variables were complementiser (present vs. absent), gender (match vs. mismatch), and their interaction. These were sum-coded (–1/+1). The models also included varying intercepts for participants and items, varying slopes for the predictors by participants and items, and random correlations among these participant/item-specific coefficients.

When the maximal model failed to converge, correlation parameters were set to zero and the model was refitted. If convergence was still not achieved, the varying-effects structure was simplified by iteratively removing the participant- or item-specific slope with the smallest estimated variance component, refitting the model after each step until convergence was obtained.

### Results

**Figure 3:**
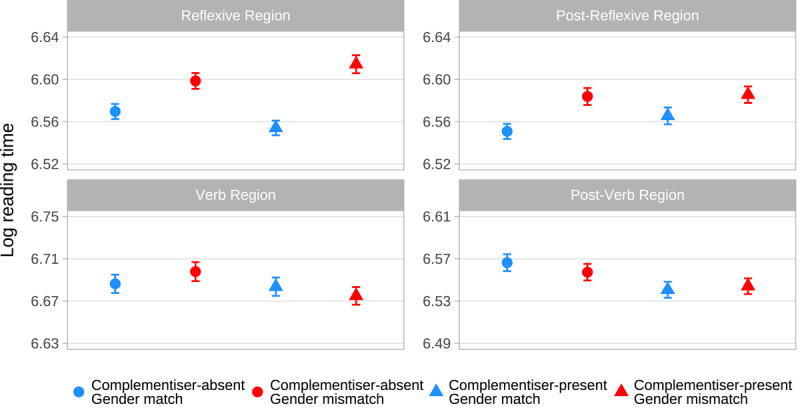
Log-transformed reading times at the (post-)reflexive and (post-)verb regions in Experiment 1. Error bars are standard errors.

**Figure 4:**
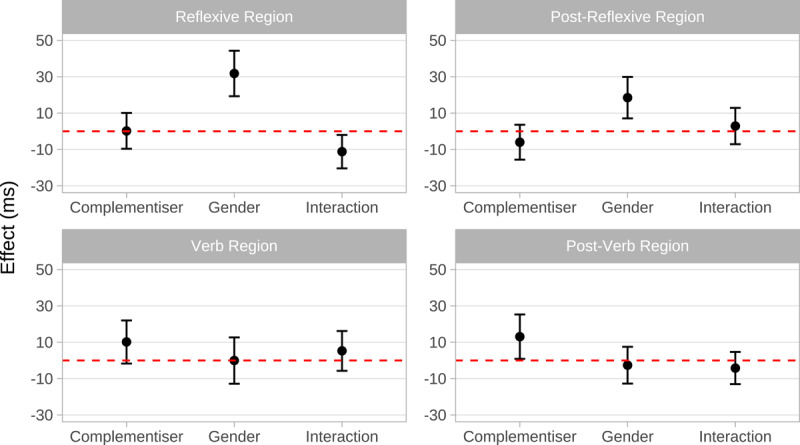
Effect estimates and 95% compatibility intervals at the (post-)reflexive and (post-)verb regions in Experiment 1.

Figure 3 presents log-transformed reading times at the four regions, and Figure 4 presents effect estimates with 95% compatibility intervals (CIs). Although the analyses were conducted on log-transformed reading times, the effect estimates reported below have been back-transformed into milliseconds for ease of interpretation.

#### Reflexive region

There was a main effect of gender (95% CI [19, 44] ms, *t* = 4.99), with longer reading times in the gender-mismatch conditions than in the gender-match conditions (i.e., a gender-mismatch effect). Although the interaction between complementiser and gender was observed (95% CI [–20, –2] ms, *t* = –2.38), a follow-up analysis examining the effect of gender within each level of complementiser showed a gender-mismatch effect in both the complementiser-absent (95% CI [6, 35] ms, *t* = 2.84) and complementiser-present (95% CI [27, 58] ms, *t* = 5.31) conditions.

#### Post-reflexive region

There was a gender-mismatch effect (95% CI [7, 30] ms, *t* = 3.17), but no interaction between complementiser and gender was observed (95% CI [–7, 13] ms, *t* = 0.58).

#### Verb region

Reading times were longer in the complementiser-absent conditions than in the complementiser-present conditions, but the evidence was weak (95% CI [–2, 22] ms, *t* = 1.68). No clear evidence was observed for the other effects (see Figure 4).

#### Post-verb region

There was a clear main effect of complementiser (95% CI [1, 25] ms, *t* = 2.11), with longer reading times in the complementiser-absent conditions than in the complementiser-present conditions (i.e., a garden-path effect). No clear evidence was observed for the other effects.

### Discussion

Experiment 1 revealed a gender-mismatch effects at the (post-)reflexive regions in both the complementiser-absent and complementiser-present conditions. This pattern suggests that semantic/categorial constraints and selectional frequency exert stronger influence than the adjunct bias, leading to adoption of the subject analysis. However, Experiment 1 also showed a garden-path effect at the post-verb region in the complementiser-absent conditions relative to the complementiser-present conditions, suggesting that the object analysis is favoured at the embedded verb due to the adjunct-induced bias.

At first glance, these two findings appear contradictory: the gender-mismatch effect indicates a preference for the subject analysis, whereas the garden-path effect indicates a preference for the object analysis. One possible reconciliation is that the subject analysis is favoured at the reflexive due to semantic/categorial constraints and selectional frequency, but the adjunct bias becomes progressively influential as the temporal adjunct is processed and ultimately overrides semantic/categorial constraints and selectional frequency (the reason why the strength of the adjunct bias may change over time is discussed in the General Discussion).

Experiment 2 was designed to test this hypothesis and to further address the first research question—whether the object analysis is maintained until embedded-verb disambiguation when semantic/categorial constraints and selectional frequency favour the subject analysis (Experiment 1 does not provide a clear answer to this question due to the influence of temporal adjuncts). Specifically, Experiment 2 examined whether a garden-path effect occurs when the temporal adjunct is removed. If the garden-path effect observed in Experiment 1 was driven by the temporal adjunct, no garden-path effect should be observed in comparable sentences without the adjunct.

## Experiment 2

Experiment 2 investigated whether a garden-path effect occurs in the absence of the temporal adjunct, using the design in (9a/b).

(9a)    *Complementiser-absent*

John hoped the boy took some time to rest from studying.

(9b)    *Complementiser-present*

John hoped that the boy took some time to rest from studying.

The sentences in (9a/b) are identical to those used in Experiment 1, except that the temporal adjunct was absent. If the garden-path effect observed in Experiment 1 was driven by the presence of the temporal adjunct, the sentences in (9a/b) should yield similar reading times at the embedded verb “*took*” (i.e., no garden-path effect).

### Participants

In Experiment 2, 136 participants were recruited via Prolific from the same participant pool as in Experiment 1. None of them had participated in Experiment 1.

### Materials

The materials consisted of 24 sets of sentences like (9a/b), 72 filler sentences, and practice items.

### Procedure and data analysis

The procedure and data analysis were identical to those in Experiment 1, except that the linear mixed-effects models did not include the predictor *gender* and the interaction term. Thus, the models included only the predictor *complementiser* (present vs. absent).

### Results

**Figure 5:**
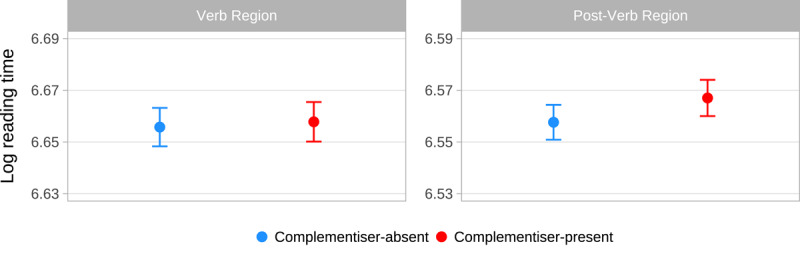
Log-transformed reading times at the (post-)verb regions in Experiment 2. Error bars are standard errors.

**Figure 6:**
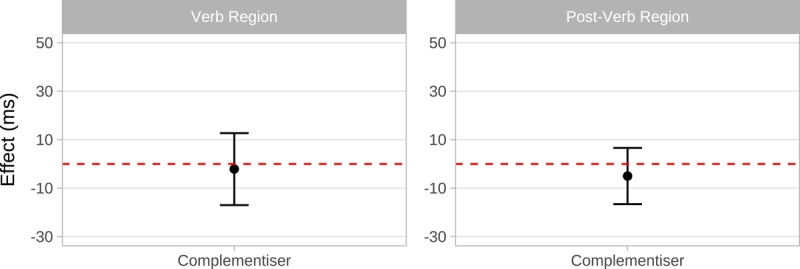
Effect estimates and 95% compatibility intervals at the (post-)verb regions in Experiment 2.

Log-transformed reading times at the verb and post-verb regions are shown in Figure 5. Effect estimates are illustrated in Figure 6.

#### Verb region

No clear effect of complementiser was observed. The 95% CI was centred around zero ([–17, 13] ms), and the *t*-value was –0.28. This pattern is consistent with the absence of a garden-path effect.

#### Post-verb region

Similarly, no garden-path effect was observed. The 95% CI was centred around zero ([–17, 7] ms), and the *t*-value was –0.85.

### Discussion

Experiment 2 showed no garden-path effect, supporting the hypothesis that the garden-path effect observed in Experiment 1 was driven by the temporal adjunct. The results further suggest that, in the absence of the adjunct bias, semantic/categorial constraints and selectional frequency lead to adoption of the subject analysis before the embedded verb is encountered.

The following section reports the final experiment (Experiment 3), which was designed to provide further and more conclusive evidence that the garden-path effect observed in Experiment 1 was due to the temporal adjunct. Specifically, Experiment 3 directly compares sentences with and without the temporal adjunct.

## Experiment 3

Experiment 3 directly compared sentences with and without the temporal adjunct, using the design in (10a–d).

(10a)    *Adjunct-present, Complementiser-absent*

John hoped the boy after washing in the bathroom took some time to rest from studying.

(10b)    *Adjunct-present, Complementiser-present*

John hoped that the boy after washing in the bathroom took some time to rest from studying.

(10c)    *Adjunct-absent, Complementiser-absent*

John hoped the boy took some time to rest from studying.

(10d)    *Adjunct-absent, Complementiser-present*

John hoped that the boy took some time to rest from studying.

Sentences (10c/d) are identical to those used in Experiment 2. Sentences (10a/b) include the temporal adjunct but omit the reflexive. If the temporal adjunct gradually becomes influential and ultimately overrides semantic/categorial constraints and selectional frequency, a garden-path effect should be observed at the embedded verb “*took*” in the adjunct-present conditions (i.e., RT_(10a)_ > RT_(10b)_), but not in the adjunct-absent conditions (i.e., RT_(10c)_ ≈ RT_(10d)_).

### Participants

For Experiment 3, 140 participants who had not participated in Experiments 1 or 2 were recruited via Prolific.

### Materials

Experiment 3 included 24 sets of experimental sentences like (10a–d), 72 filler sentences, and practice items.

### Procedure and data analysis

The procedure and data analysis were similar to Experiment 1, with one difference: the linear mixed-effects models did not include *gender* and its interaction with complementiser. Instead, the models included *adjunct* (present vs. absent) and its interaction with complementiser.

### Results

**Figure 7:**
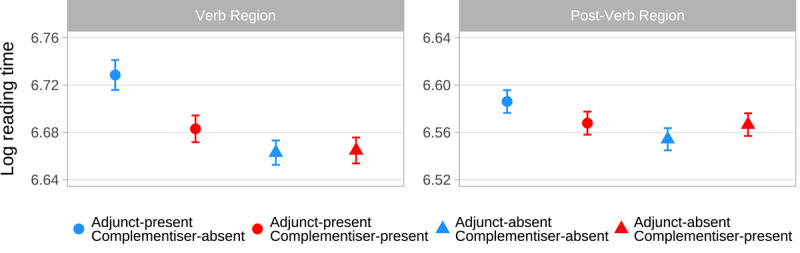
Log-transformed reading times at the (post-)verb regions in Experiment 3. Error bars are standard errors.

**Figure 8:**
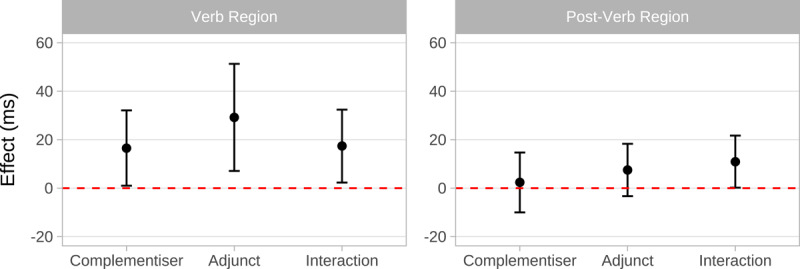
Effect estimates and 95% compatibility intervals at the (post-)verb regions in Experiment 3.

Log-transformed reading times at the verb and post-verb regions are shown in Figure 7, and effect estimates with 95% CIs are shown in Figure 8.

#### Verb region

There was a main effect of adjunct (95% CI [7, 51] ms, *t* = 2.59), with longer reading times in the adjunct-present conditions than in the adjunct-absent conditions. This effect likely reflects increased processing cost associated with the greater distance between the critical NP (“*the boy*”) and the embedded verb (“*took*”) in adjunct-present sentences (“*John hoped that **the boy** after washing in the bathroom **took**…*”) relative to adjunct-absent sentences (“*John hoped that **the boy took**…*”; see Fujita & Cunnings, 2024; Gibson & Warren, 2004; Keine, 2020). There was also a main effect of complementiser (95% CI [1, 32] ms, *t* = 2.09), indicating longer reading times in the complementiser-absent conditions than in the complementiser-present conditions (i.e., a garden-path effect). Crucially, there was an adjunct × complementiser interaction (95% CI [2, 32] ms, *t* = 2.26). A follow-up analysis examining the effect of complementiser within each level of adjunct revealed a garden-path effect in the adjunct-present conditions (95% CI [12, 56] ms, *t* = 3.04), but not in the adjunct-absent conditions (95% CI [–22, 20] ms, *t* = –0.07).

#### Post-verb region

The post-verb region showed a similar pattern. There was an interaction (95% CI [0.2, 22] ms, *t* = 1.99), suggesting a weak garden-path effect in the adjunct-present conditions (95% CI [–3, 29] ms, *t* = 1.57) but no garden-path effect in the adjunct-absent conditions (95% CI [–25, 8] ms, *t* = –1.04).

### Discussion

Experiment 3 showed a garden-path effect at the embedded verb when the temporal adjunct was present but not when it was absent. This pattern is consistent with Experiments 1 and 2 and provides direct evidence that the temporal adjunct overrides semantic/categorial constraints and selectional frequency as it is processed. The General Discussion considers the implications of Experiments 1–3 for mechanisms of real-time sentence processing.

## General Discussion

The present study aimed to address two research questions: (i) whether the object analysis is maintained until the embedded verb appears when semantic/categorial constraints and selectional frequency favour the subject analysis; and (ii) how adjunct bias interacts with semantic/categorial constraints and selectional frequency during real-time ambiguity resolution.

In Experiment 1, a gender-mismatch effect was observed at the reflexive in both complementiser-present and complementiser-absent sentences (e.g., “*John hoped (that) **the boy/girl** after washing **himself** in the bathroom took…*”). This pattern suggests that the processor favours the subject analysis at the reflexive due to the influence of semantic/categorial constraints and selectional frequency. However, Experiment 1 also revealed a garden-path effect, indicating that the object analysis is favoured at the disambiguating verb. Although these findings may appear contradictory, Experiments 2 and 3 clarify their source. Specifically, a garden-path effect emerged only when the temporal adjunct was present, and disappeared when it was absent. These results indicate that the temporal adjunct is responsible for the later difficulty observed at the embedded verb.

Experiments 1–3 suggest that semantic/categorial constraints and selectional frequency strongly bias parsing towards the subject analysis before the embedded verb is encountered, in line with the constraint-based model (e.g., Garnsey et al., 1997). The temporal adjunct biases parsing towards the object analysis, but this bias does not immediately lead the processor to favour the object analysis: semantic/categorial constraints and selectional frequency initially exert stronger influence. However, as the temporal adjunct is processed, its bias becomes stronger, eventually overriding semantic/categorial constraints and selectional frequency, leading to a garden-path effect at the embedded verb.

The claim that adjunct bias becomes progressively influential aligns with the commitment principle (Crocker, 1996), according to which the longer the processor remains committed to a particular analysis, the stronger that commitment becomes. Evidence for the commitment principle comes from findings that prolonged involvement in a misanalysis makes it more difficult to recover from that misanalysis (e.g., Ferreira & Henderson, 1991, 1998; Fodor & Ferreira, 1998; Fujita & Cunnings, 2020; Tabor & Hutchins, 2004). This phenomenon is sometimes referred to as a digging-in effect (Tabor & Hutchins, 2004).

For example, consider the following locally ambiguous sentence: “*While Mary dressed the girl caught a butterfly*”. In this sentence, the locally ambiguous NP “*the girl*” is initially misanalysed as the direct object of “*dressed*” (as in “*After Mary dressed the girl,…*”). Revision is required at the verb “*caught*”, where “*the girl*” needs to be interpreted as the matrix subject, as in “*After Mary dressed, the girl caught…*” (e.g., L. Frazier & Rayner, 1982; Fujita & Cunnings, 2021; Fujita & Yoshida, 2026a; Slattery et al., 2013; Sturt et al., 1999). Previous work shows that revision from the object to the subject becomes more difficult (e.g., a garden-path effect becomes larger) as the locally ambiguous NP becomes longer (e.g., “*While Mary dressed the girl **who was in the garden** caught a butterfly*”; e.g., Ferreira & Henderson, 1991).

The present proposal can be understood in similar terms: the longer the temporal adjunct is processed, the more strongly it biases the processor towards the syntactic structure it supports (i.e., the object analysis), given that adjunct bias is position-dependent. This leads to a garden-path effect at the embedded verb.

**Figure 9:**
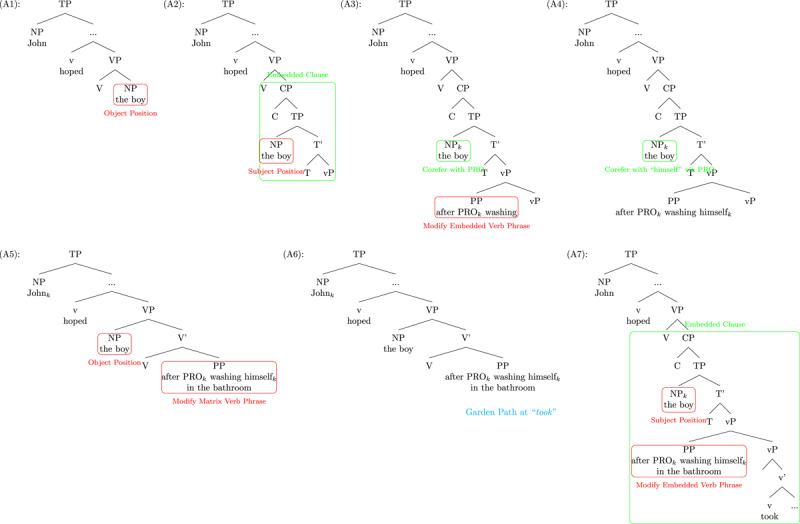
A proposed parsing process in the complementiser-absent sentence: “John hoped the boy after washing himself in the bathroom took…”.

In summary, based on the results of Experiments 1–3 and previous research, we can hypothesise the following parsing process:

1. When the critical NP (“*John hoped **the boy**…*”) is encountered during real-time processing, it is initially parsed as the direct object of the matrix verb (Pickering et al., 2000; A1; see Figure 9). However, the processor rapidly abandons this analysis and adopts the subject analysis, driven by semantic/categorial constraints and selectional frequency (A2).
2. When the preposition “*after*” is encountered and PRO is identified (“*John hoped the boy **after PRO washing**…*”), the temporal adjunct introduces a bias towards the object analysis. At this point, however, the processor still favours the subject analysis because semantic/categorial constraints and selectional frequency exert a stronger influence. Consequently, coreference is established between “*the boy*” and PRO (A3).
3. When the reflexive is encountered (“*John hoped the boy after PRO washing **himself**…*”), it is interpreted as coreferential with PRO, which corefers with “*the boy*” (A4). If the embedded subject is a feminine NP (“*John hoped **the girl** after washing himself…*”), a gender-mismatch effect arises.
4. As the temporal adjunct is processed further (“*John hoped the boy after washing himself in the bathroom…*”), its bias towards the object analysis strengthens and eventually override semantic/categorial constraints and selectional frequency. As a result, the object analysis is adopted near the end of the temporal adjunct (A5), leading to a garden-path effect at the embedded verb (A6).
5. Upon encountering the embedded verb (“*John hoped the boy after washing himself in the bathroom **took**…*”), “*the boy*” is revised as the embedded subject (A7).

This parsing process could in principle be modelled within a range of approaches that assume that multiple sources of information dynamically influence real-time sentence processing (e.g., Crocker, 2010; Crocker & Brants, 2000; Hale, 2001; Levy, 2008; Tabor & Tanenhaus, 1999). For example, expectation-based approaches (Hale, 2001; Levy, 2008) characterise processing difficulty in terms of surprisal (Hale, 2001), a probabilistic metric derived from the distribution over possible analyses. The surprisal of a word (*w*) is defined as the negative log probability of that word given context (*c*): –log*P*(*w*|*c*). Accordingly, surprisal increases as the conditional probability decreases, corresponding to greater processing difficulty. Because this probability distribution is shaped by multiple sources of information and updated incrementally with each incoming word, expectation-based approaches could account for the present findings.

In the present study, the proposed parsing process is modelled within the constraint-based framework (McRae et al., 1998; Spivey-Knowlton & Tanenhaus, 1998).

Recall that, for the complementiser-absent sentences tested in Experiment 1 (“*John hoped the boy/girl after washing himself in the bathroom took…*”), the constraint-based model predicts that the subject analysis is ultimately favoured at the critical NP (“*the boy/girl*”). Consistent with this prediction, Experiment 1 showed that the subject analysis is favoured at the reflexive (A4 in Figure 9). However, Experiment 1 (and Experiment 3) also indicated that the object analysis is adopted near the end of the adjunct (A5). Above, I proposed that this shift occurs because the adjunct bias towards the object analysis becomes stronger than semantic/categorial constraints and selectional frequency as the temporal adjunct is processed.

One way to incorporate this commitment-related influence into the constraint-based model is to use activation weights (i.e., $i_{a}=\Sigma_{b}\;w_{b}S_{\text{b,a}}^{\prime }$ ). Specifically, when the preposition “*after*” is encountered during processing (“*John hoped the boy after …*”), semantic/categorial constraints, selectional frequency, and adjunct bias are assumed to contribute equally (i.e., semantic/categorial constraint = 1/3, selectional frequency = 1/3, adjunct bias = 1/3). As the processing of the temporal adjunct proceeds, however, adjunct bias receives greater weight than the other sources (e.g., semantic/categorial constraint = 3/10, selectional frequency = 3/10, adjunct bias = 4/10).

I simulated the incremental parsing process in R. The simulation code is available on the OSF website (https://osf.io/4mkwf/). The model focused on three sources of bias: semantic/categorial constraints, selectional frequency, and adjunct bias. Their weights are summarised in Table 1. The selectional-frequency bias was derived from previous research (Trueswell et al., 1993), and the semantic/categorial bias was based on the acceptability judgements reported in the present study and in previous work (Garnsey et al., 1997; Qian et al., 2019). Based on the continuation data sampled using GPT-2 (reported in the Introduction), the adjunct bias was assumed to strongly favour the object analysis.

**Table 1:** Normalised strengths of the three biases and bias weights in the sentence: “John hoped the boy after washing himself in the bathroom…”.


BIAS	SUBJECT ANALYSIS	OBJECT ANALYSIS	WEIGHT (AFTER)	WEIGHT (HIMSELF IN)	WEIGHT (THE BATHROOM)

Semantic/Categorial Constraint	0.76	0.24	1/3	3/10	2/10

Selectional Frequency	0.94	0.06	1/3	3/10	2/10

Temporal Adjunct	0.02	0.98	1/3	4/10	6/10


At the beginning of the temporal adjunct, the three sources of bias were equally weighted. However, as the simulation progressed towards the end of the temporal adjunct, the adjunct bias was weighted more heavily than the others. The model further assumed that each word in the temporal adjunct incrementally strengthened the object-analysis bias (i.e., at each word, the activation level of the adjunct bias was reset to the original value).

**Figure 10:**
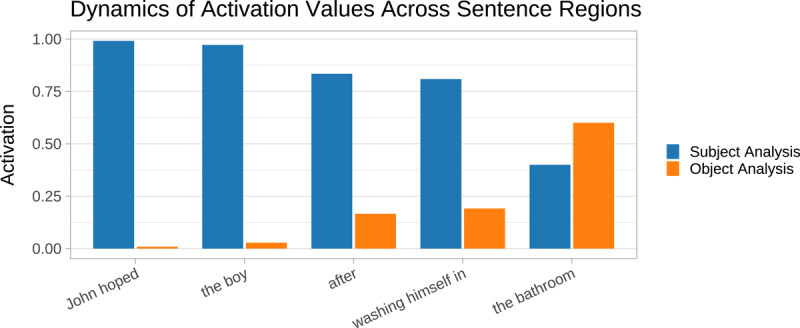
Constraint-based modelling result. Activation values shown are those that reach the thresholds.

Figure 10 displays the modelling results. As shown, the model captures the parsing process observed in this study. Specifically, at the critical NP (“*the boy/girl*”), the model predicts a strong preference for the subject analysis, consistent with the present findings.^2^ When the preposition “*after*” appears, the adjunct bias begins to influence parsing. At the reflexive region, the subject analysis still shows higher activation than the object analysis. Towards the end of the temporal adjunct, however, the adjunct bias receives greater weight, resulting in higher activation for the object analysis than for the subject analysis. This shift mirrors the observed reading-time pattern, in which a garden-path effect emerged at the embedded verb following the temporal adjunct. Taken together, the modelling suggests that predicting the parser’s behaviour during the real-time resolution of complement ambiguity may require assuming that parsing is dynamically influenced by multiple interacting biases.

## Conclusion

This study reported three experiments examining how semantic/categorial constraints, selectional frequency, and temporal adjuncts influence the real-time resolution of complement ambiguity. Semantic/categorial constraints and selectional frequency favoured the subject analysis, whereas temporal adjuncts biased parsing towards the object analysis. The results revealed that semantic/categorial constraints and selectional frequency lead the processor to adopt the subject analysis before the embedded verb appears. However, there was evidence that the adjunct bias gradually strengthens during processing and ultimately overrides semantic/categorial constraints and selectional frequency. Computational modelling further supports this interpretation, suggesting that the adjunct bias can dynamically shift activation in favour of the object analysis over time. Together, these findings underscore the importance of considering sentence processing as a dynamic system in which multiple sources of information interact and vary in strength throughout sentence comprehension.

## Data Availability

Data, analysis code experimental materials, and simulation code are available at https://osf.io/4mkwf/.

## Footnotes

As a reviewer pointed out, Pickering et al. (2000) included an adjunct immediately after the locally ambiguous NP, as in “*The young athlete realised her potential one day might…*”. The present study differs from Pickering et al. in that it examines the influence of temporal adjuncts, which are expected to bias parsing strongly towards the object analysis. In addition, whereas Pickering et al. examine initial analysis at the locally ambiguous NP, the present study focuses on ambiguity resolution beyond the initial analysis. To test whether expressions such as “*one day*” introduce a weaker bias than temporal adjuncts, I generated 100 continuations for the sentence fragment “*The union leader implied the raise one day…*”. The results showed that object-analysis continuations were substantially less frequent (45/100), suggesting that expressions of this type exert a weaker bias than the temporal adjuncts used in the present experiments.

This modelling does not capture Pickering et al.’s (2000) finding that the processor initially attempts to analyse the critical NP (“*the boy*”) as the direct object of the matrix verb, even when semantic constraints and selectional frequency favour the subject analysis. Capturing this pattern would require positing an additional factor that strongly biases parsing towards the object analysis at the critical NP, but only during an early stage of processing (L. Frazier, 1987; L. Frazier & Clifton, 1996).
